# NF‐YA promotes the cell proliferation and tumorigenic properties by transcriptional activation of SOX2 in cervical cancer

**DOI:** 10.1111/jcmm.15777

**Published:** 2020-09-20

**Authors:** Wen‐Ting Yang, Qian Feng, Hong‐Mei Ma, Dan Lei, Peng‐Sheng Zheng

**Affiliations:** ^1^ Department of Reproductive Medicine The First Affiliated Hospital of Xi’an Jiaotong University Shaanxi, Xi’an China; ^2^ Key Laboratory of Environment and Genes Related to Diseases Ministry of Education of the People's Republic of China Shaanxi, Xi’an China

**Keywords:** NF‐YA, SOX2, cervical cancer, cancer stem cell

## Abstract

NF‐YA is considered as a crucial regulator for the maintenance of cancer stem cell (CSC) and involved in various types of malignant tumours. However, the exact function and molecular mechanisms of NF‐YA in the progression of cervical cancer remains poorly understood. Here, the expression of NF‐YA detected by immunohistochemistry was gradually increased from normal cervical tissues, to the high‐grade squamous intraepithelial lesions, and then to cervical cancer tissues. NF‐YA promoted the cell proliferation and tumorigenic properties of cervical cancer cells as well as tumorsphere formation and chemoresistance *in vitro*. The luciferase reporter assay combined with mutagenesis analyses and Western blotting showed that NF‐YA trans‐activated the expression of SOX2 in cervical cancer. Furthermore, quantitative chromatin immunoprecipitation (qChIP) and electrophoretic mobility shift assay (EMSA) confirmed that NF‐YA protein directly bound to the CCAAT box region located upstream of the SOX2 promoter. Together, our data demonstrated that NF‐YA was highly expressed in cervical cancer and promoted the cell proliferation, tumorigenicity and CSC characteristic by trans‐activating the expression of SOX2.

## 1 INTRODUCTION

Cervical cancer is the fourth most common malignancy among women and the seventh overall, with estimated 527,600 new cases and 265,700 deaths worldwide in 2018.^1^ It is well established that the high‐risk human papillomavirus (HPV) is the major risk factor for cervical carcinogenesis, but the infections by HPV are mostly transient and cleared within a few months. Additionally, the genetic alterations and epigenetic modifications are also involved in the initiation and progression of cervical cancer. Recently, extensive studies have shown that the stem cell‐associated transcription factors, such as SOX2,^2^ SOX9,^3^ NANOG,^4^ KLF4,^5^ LGR5,^6^, ^7^ UTF1,^8^ OCT4^9^ and DAX1,^10^ are anomaly modulated and functionally alter signalling pathways during cervical cancer pathogenesis.

Cancer stem cell (CSC) or tumour initiating cell, a small sub‐population cells, is hypothesized to be involved in tumour growth, metastasis and recurrence.^11^ Over the past decade, increasing numbers of studies have been carried out to identify and isolate CSC from several types of human cancers depending on a variety of different cell surface markers, including CD133, CD44, CD166 and CD24 among others.^12^ However, the surface markers are of instability and scarcity to isolate CSC in solid tumours. Thus, other markers, such as nuclear transcription factors,^13^ side populations,^14^ sphere formation and aldehyde dehydrogenase (ALDH) activity,^15^ have been widely explored. In our previous study, the cervical CSC has been isolated and identified by the nuclear transcription factor SOX2^16^ and cytoplasm ALDH1.^17^ However, the exact mechanism and key genes involved in the maintenance and regulation of cervical CSC have not been clearly revealed.

NF‐Y (nuclear transcription factor‐Y, also known as CBF, CCAAT‐binding factor), a heterodimeric protein complex, is a ubiquitously expressed trimetric transcription factor comprising the subunits (NF‐YA, NF‐YB and NF‐YC).^18^ NF‐YA is required for the complex assembly and sequence‐specific DNA binding to CCAAT box. It is reported that NF‐YA functions as an oncogene or suppressor through the mechanism of cell proliferation, metastasis and tumorigenicity.^19^, ^20^, ^21^, ^22^ The recent study has identified NF‐YA as a CSC marker in hepatocellular cancer, oral cancer and embryonic cancer.^20^, ^23^, ^24^, ^25^ It was shown that NF, as a transcription factor, regulates the expression of several human SOX genes, including SOX3,^26^ SOX9^27^ and SOX18,^28^ by direct binding to CCAAT boxes within promoters of target genes and by making complex interplay with other factors involved in transcription regulation of human SOX genes.

Here, our study demonstrated that NF‐YA was up‐regulated during the progression of cervical cancer, which was essential for the promotion of cell proliferation, tumorigenicity and stemness properties by transcriptional activation of SOX2 protein in cervical cancer.

## 2 MATERIALS AND METHODS

### 2.1 Human tissue specimens

Normal cervix (NC, N = 31) tissues, high‐grade squamous intraepithelial lesions (HSIL, N = 30) tissues and squamous cervical cancer (SCC, N = 67) tissue samples were collected from the First Affiliated Hospital of Xi’an Jiaotong University from 2012 to 2017. None of the patients had received chemotherapy, immunotherapy or radiotherapy before the specimens’ collection. The histological classification and clinical staging were carried out in accordance with the International Federation of Gynecology and Obstetrics classification system. Normal cervix tissues were obtained from the patients with hysteromyoma but without HPV infection and dysplasia. The institutional review board named as Ethics Committee of Medical School of Xi’an Jiaotong University in Shannxi, China, approved the population study, and the informed consents were approved prior to specimen collection with the authorization number of 2019GKJ‐244.

### 2.2 Immunohistochemistry and immunocytochemistry

Four‐μm thick sections of formalin‐fixed paraffin‐embedded tissue samples on the slides, including NC, HSIL and SCC, were successively deparaffinized, rehydrated and then pre‐treatment with 10mM sodium citrate antigen retrieval buffer (pH 6.0) in a steam pressure cooker. After treated with 3% H_2_O_2_, following the sections were incubated with antibodies, including human anti‐goat SOX2 (1:200; #sc‐17320, Santa Cruz), anti‐mouse NF‐YA (1:400; #sc‐17753, Santa Cruz) and anti‐mouse Ki‐67 (1:200; #sc‐23900, Santa Cruz). After overnight at 4°C, the sections were washed and incubated with streptavidin‐peroxidase complex for 30 minutes at room temperature, and then immunostaining was performed using 0.05% 3’3‐diaminobenzidine (DAB) followed by counterstaining with haematoxylin.

The expression of NF‐YA stained by IHC was valued through the immunoreactivity score (IRS). The percentage of NF‐YA‐positive cells was divided into 5 score ranks: <10% (0), 10% to 25% (1), 25% to 50% (2), 50% to 75% (3) and> 75% (4). The intensity of NF‐YA staining was divided into 4 score ranks: no staining (0), light brown (1), brown (2) and dark brown (3). The positive specimens of NF‐YA staining were determined by IRS (intensity score × quantity score). The overall score of ≤ 3 was defined as negative, >3 but ≤ 6 as weak positive, and> 6 as strong positive. Two different pathologists evaluated all the specimens in a blinded manner.

The expression of NF‐YA protein was also detected by immunocytochemistry experiment in cervical cancer cells. In brief, cells cultured on coverslips were fixed with 4% paraformaldehyde for 30 minutes, permeabilized with 0.2% Triton X‐100 for 15 minutes at room temperature and then incubated with the primary antibodies described above.

### 2.3 Cervical cancer cell lines

The human cervical cancer cell lines, including SiHa, HeLa, C33A, CaSki and HT‐3, were obtained from the American Type Culture Collection (ATCC). SiHa, HeLa and C33A cells were cultured in DMEM (Dulbecco’s modified Eagle medium‐high glucose, Sigma‐Aldrich), CaSki cells were cultured in RPMI1640 (Sigma‐Aldrich), and HT‐3 cells were cultured in McCoy’s 5A Medium^10^, ^27^ all supplemented with 10% FBS (foetal bovine serum, Invitrogen, Carlsbad, CA). All the cells were maintained at 37°C in an atmosphere containing 5% carbon dioxide.

### 2.4 Vector construction and transfection

Human full‐length NF‐YA (NM_002505.5) cDNA was amplified by RT‐PCR (reverse transcription‐polymerase chain reaction) using mRNA extracted from cervical cancer cell line HeLa. The cDNA fragment was subsequently cloned into pIRES2‐AcGFP1‐Neo (Clontech), to generate the recombinant pIRES2‐AcGFP1‐Neo‐NF‐YA plasmid. The empty plasmid pIRES2‐AcGFP1‐Neo and overexpression plasmid pIRES2‐AcGFP1‐Neo‐NF‐YA were transfected into SiHa and C33A cells using Lipofectamine 2000 reagent (Invitrogen) according to the manufacturer’s instructions. Successful transfection was verified as green fluorescence under fluorescence microscope. Then, G418 with a concentration of 1g/L was added for screening. Stable transfected SiHa and C33A cells were treated with G418 (Calbiochem) for ~ 3 weeks, the clones expressing neomycin resistance steadily were obtained and expanded, and the expression of NF‐YA protein was identified by Western blotting. NF‐YA‐overexpressing cell clones (SiHa‐NF‐YA and C33A‐NF‐YA) and control cell clones (SiHa‐GFP and C33A‐GFP) were maintained as monoclonal cells treated with G418 with a concentration of 1 g/L all the time.

ShRNA plasmid targeting SOX2 (shSOX2) and scrambled control shRNA (shNC) were purchased from GenePharma. NF‐YA overexpression SiHa cells were stably transfected by shSOX2 and shNC plasmids using the Lipofectamine 2000. The knockdown efficiency was evaluated using Western blotting analysis.

### 2.5 Western blotting

Cells were lysed in NP‐40 buffer (15 mL 5 m NaCl, 2.5 ml NP‐40, 25 mL 1 m Tris‐HCl (pH 7.5), 1.05g NaF for a total of 500 mL) containing protease inhibitor cocktail (Complete Mini; Roche Diagnostics). Protein concentrations were determined using the bicinchoninic acid assay kit (Pierce Chemical Corporation). A total of 30μg protein was performed as previously described by Western blotting assays.^10^ The primary antibodies mouse anti‐human NF‐YA (1:1000, #sc‐17753, Santa Cruz), goat polyclonal anti‐human SOX2 (1:500, #sc‐17320, Santa Cruz) and mouse anti‐human GAPDH (1:1000, #sc‐32233, Santa Cruz) were performed at 4℃ overnight. A horseradish peroxidase‐conjugated anti‐mouse or anti‐goat IgG (Thermo Fisher Scientific) as the secondary antibodies was incubated at room temperature for 1 hour. The signals were then detected by enhanced chemiluminescence reagent (Millipore).

### 2.6 *In vivo* tumour formation assays

Cells were injected into the subcutaneous on the dorsum of the female nude mice (4‐6 weeks old) with a mixture of 1:1 Matrigel (Becton Dickinson) to assess the tumour formation properties *in vivo*. A total of 18 mice were randomly divided into 2 groups in triplicates for three for every pair of cells: SiHa‐NF‐YA, SiHa‐GFP, C33A‐NF‐YA and C33A‐GFP. Engrafted mice were inspected every three days by visual observation and palpation for the appearance of tumours, and mice were killed when the maximum tumour volume was closed to 2 cm^3^. The tumour volume (V) was determined by the length (a) and width (b) as V = ab^2^/2. The tumour‐free period was determined by the time of palpable tumour formation. The experimental protocols were evaluated and approved by the Animal Care and Use Committee of the Medical School of Xi’an Jiaotong University with the authorization number of 2019GKJ‐244. A portion of each tumour tissue was fixed in 10% formaldehyde and embedded in paraffin for IHC analysis.

### 2.7 Cell growth and viability assays

Cell growth assay was performed for 7 days. In brief, 2 × 10^4^ cells were cultured in 35‐mm culture dishes, harvested every day and then the living cells dyed by trypan were counted using a haemocytometer under the light microscope.

Additionally, cell viability assay was assessed according to a standard protocol using MTT dye (3‐(4,5‐dimethylthiazole‐yl)‐2,5‐diphenyltetrazolium bromide, Sigma‐Aldrich). Following the manufacturer’s instructions, 1 × 10^3^ cells were planted in 96‐well plate and 20 µL of MTT solution with the concentration of 5 mg/mL was added to 200 µL of the culture media every day for one week. The plates were then incubated for 4 hours at 37°C, and the optical density was measured at 490 nm.

### 2.8 The detection of chemoresistance

For the chemotherapy drug resistance assays, cells were cultured in 96‐well plates at a density of 10^4^ cells/well and allowed to recover overnight before initiating drug treatments. The cell viability with MTT assay was measured when the cells were exposed in various concentrations of cisplatin (0, 3, 6, 12 or 24 µg/mL) and 5‐Fu (5‐Fluorouracil, 50, 100 or 200 µg/mL) for 24 hours. Then, the value of IC50 for drugs was calculated. Additionally, the cells were exposed to a constant concentration of cisplatin (6 µg/mL) and 5‐Fu (100 µg/mL) for 24, 48 or 72 hours, and the cell viability was measured.

Also, cells (1 × 10^5^) cultured in 35‐mm dish were treated with cisplatin (6 µg/mL) and 5‐Fu (5‐Fluorouracil, 100 µg/mL) for 72 hours, and then, the cell viability was determined by Giemsa staining. For Giemsa staining, briefly, cells were washed with PBS and fixed with formaldehyde for 30 minutes and washed again before incubation with Giemsa staining solution (Sigma). After 30 minutes of staining, cells were washed and allowed to dry. The viability cells were counted in 10 random high‐power field.

### 2.9 Cell cycle detected by FACS

A cell cycle analysis was performed using fluorescence‐activated cell sorting (FACS) (Becton Dickinson) according to the manufacturer's protocol. A number of 10^6^ cells were harvested and fixed in 70% ethanol overnight at 4°C. At 30 minutes before FACS analysis, the cells were treated with RNase A (20 μg/mL) and then stained with 50μg/mL propidium iodide (Sigma‐Aldrich). Cell cycle distribution was analysed with the FACScalibur flow cytometer using the ModFit LT software (Becton Dickinson).

### 2.10 Tumorsphere formation assay

Cells with the density of 200 cells per well in 24‐well ultra‐low attachment plates or the density of 1 cell per well in 96‐well plates in triplicate were maintained in the serum‐free medium with DMEM/F12, containing N2 and B27 supplements (Invitrogen), 20 ng/mL human recombinant epidermal growth factor (EGF) and 20 ng/mL basic fibroblastic growth factor (bFGF; PeproTech Inc., Rocky Hill, NJ). The number of tumorspheres generated within 2 weeks was counted and calculate the percentage of sphere‐forming. For serial tumorsphere formation assays, the spheres were harvested, disaggregated with 0.25% trypsin/EDTA, filtered through a 40‐μm mesh and re‐plated as described above.

### 2.11 SOX2 promoter reporters

To characterize the transcriptional effects of mutations in the SOX2 promoter, we constructed the SOX2 promoter reporters by cloning the 5’ UTR (110bp) and 3’ UTR (1264) regions of the SOX2 promoter though 5' and 3' UTRs on both sides of the luciferase gene using an In‐Fusion PCR Cloning Kit (Takara Bio Inc) based on the pGL3 backbone (#E1751, Promega Corporation), that including PGL3‐pSox2‐4650 + 1828, PGL3‐pSox2‐3081 + 1828, PGL3‐pSox2‐1185 + 1828 and PGL3‐pSox2 mini + 1828.^16^ Then, the predictive domains bound by NF‐YA were mutated by PCR. The primers used for constructing the deletions and mutations were listed in Table S1.

### 2.12 Dual‐luciferase reporter assay

NF‐YA overexpressed SiHa, C33A cells and the control cells, respectively, with the number of 5 × 10^4^ planted in the 24‐well plate dish were transiently co‐transfected using Lipofectamine^TM^2000 (Life Technologies corporation) with the SOX2 promoter luciferase reporters and pTK‐RL plasmids. After 48 hours post‐transfection, the activity of firefly and Renilla luciferase was detected employing the Dual‐luciferase assay kit (Promega). The SOX2 promoter luciferase reporter activity was presented as the relative ratio of firefly luciferase activity to Renilla luciferase activity. The specific activity was displayed as the fold change of the experimental group versus the control group. All experiments were performed in triplicate.

### 2.13 Chromatin immunoprecipitation (ChIP)

ChIP assay was performed according to the manufacturer’s protocol for the EZ‐ChIP^TM^ assay kit (#17‐371, Millipore). The amount of precipitated DNA was calculated as 10% of the input sample in triplicate. In brief, 20 μL NF‐YA antibody (#sc‐17753, Santa Cruz) and 40 μL SOX2 antibody (#sc‐17320, Santa Cruz) were validated to immunoprecipitate the chromatin DNA cross‐linked complex with 1% formaldehyde with the normal mouse IgG and Histone H3 rabbit monoclonal antibodies as the negative and positive control, respectively. Regions of interest were amplified from precipitated samples by real‐time PCR. Each sample was assayed in triplicate, and the amount of precipitated DNA was calculated as a percentage of the input sample. The primers used in quantitative ChIP assays are as follows: Forward: 5’‐AGGGGATACAAAGGTTTCTCAGTG‐3’, Reverse: 5’‐GGCTGTCAGGGAATAAATGGG‐3’.

### 2.14 Electrophoretic mobility shift assay (EMSA)

EMSA was performed using the LightShift Chemiluminescent EMSA Kit (Thermo Fisher Scientific). Briefly, the nuclear protein lysate from NF‐YA overexpressed SiHa and C33A cells was incubated at room temperature in 10 mM Tris‐HCl, 50 mM KCl, 2.5% (vol/vol) glycerol, 5 mM MgCl_2_, 50 ng/μL poly (dI‐dC) and 0.05% (vol/vol) NP‐40 binding buffer at pH 7.5. After 10 minutes, 20 fmol of the biotin‐labelled DNA fragment containing NF‐YA binding sites (CCAAT/ATTGG) was added to the equilibrated, unclear protein and then incubated at room temperature for 20 minutes. The samples were resolved on a 5% non‐denaturing polyacrylamide gel prepared in 45 mM Tris‐borate and 1mM EDTA (TBE) buffer. The specimens were electrotransferred onto a 0.45‐μm Immobilon‐NY + nylon membrane (Millipore) at 350 mA for 40 minutes on ice. Then, the membrane was cross‐linked using an ultraviolet cross‐linker (CL‐1000 with the shortwave UV of 254 nm). The blots were developed using a Chemiluminescent Nucleic Acid Detection Module Kit (Thermo Fisher Scientific).

### 2.15 TCGA data acquisition

RNAseq data were acquired using TCGA (The Cancer Genome Atlas) database by cervical squamous cell CESC patients (N = 306) matched with the TCGA normal data (N = 13). According to TCGA publication guidelines, these mRNA sequencing data have no restrictions on publication, and no additional approval by an ethics committee was required (http://cancergenome.nih.gov/publications/publicationguidelines).

### 2.16 Statistical analysis

Statistical analyses were performed based on the software GraphPad Prism 5.01. In detail, when compared between two groups, two‐tailed Student’s *t* test was applied. To examine differences among 3 groups, an ANOVA was performed. A *P* value of < .05 was regarded as statistically significant.

## 3 RESULTS

### 3.1 Expression of NF‐YA in normal cervix and various cancerous cervical lesions

**Figure 1:**
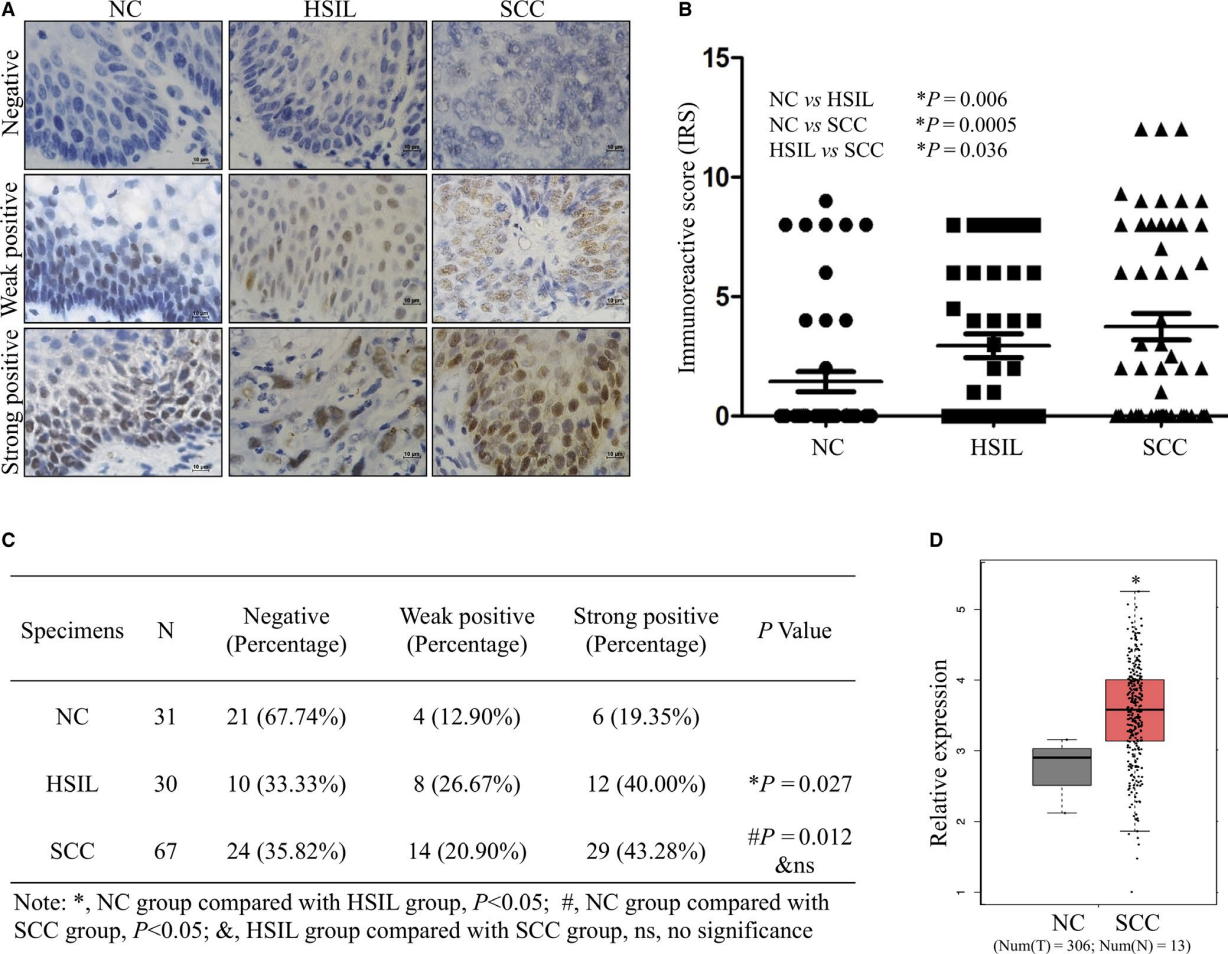
Expression of NF‐YA protein in paraffin‐embedded cervical tissue specimens. (A) Immunohistochemical staining for NF‐YA protein in 31 NC (normal cervical), 30 HSIL (high‐grade squamous intraepithelial lesion) and 67 SCC (squamous cervical carcinoma) specimens was performed, and then, the expression was divided into three staining levels: negative, weak positive and strong positive. Representative images of the NF‐YA staining were shown (scale bar: 10µm). (B) Semi‐quantitative analysis of the positive rate of NF‐YA expression is illustrated. (C) The percentage of negative, weak positive and strong positive NF‐YA expression was showed. (D) The data set from GEPIA (Gene Expression Profiling Interactive Analysis) repository (http://gepia.cancer‐pku.cn/) showed the expression of NF‐YA mRNA in cervical cancer tissues and normal cervical tissues. Error bars represent S.D.* *P* < 0.05

Firstly, we evaluated the expression of NF‐YA protein in human NC, HSIL and SCC samples by IHC. The NF‐YA staining located in the nucleus of positive cells in various cervical tissues and representative negative, weak positive and strong positive samples was observed (Figure 1A). According to the established scoring criteria, the immunoreactive score (IRS) of NF‐YA protein in SCC (N = 67) with 4.88 ± 0.47 showed much higher than that in NC (1.65 ± 0.31, N = 31) and HSIL (3.68 ± 0.51, N = 30), indicating the significant differences between HSIL and SCC samples as well as SCC and NC samples (Figure 1B, NC vs. HSIL, *P* < 0.05; NC vs. SCC, *P* < 0.05; HSIL vs. SCC, *P* < 0.05). The strong and weak positive percentage (IRS> 6) were both gradually increased from NC to HSIL and SCC (Figure 1C, *P* < 0.05). Additionally, the mRNA expression of NF‐YA was significantly increased in SCC comparing with that in NC tissues analysed by TCGA database (Figure 1D, *P* < 0.05). These results above suggested that NF‐YA might promote the progression of cervical cancer.

### 3.2 NF‐YA promotes the tumorigenic property of cervical cancer cells *in vivo*

**Figure 2:**
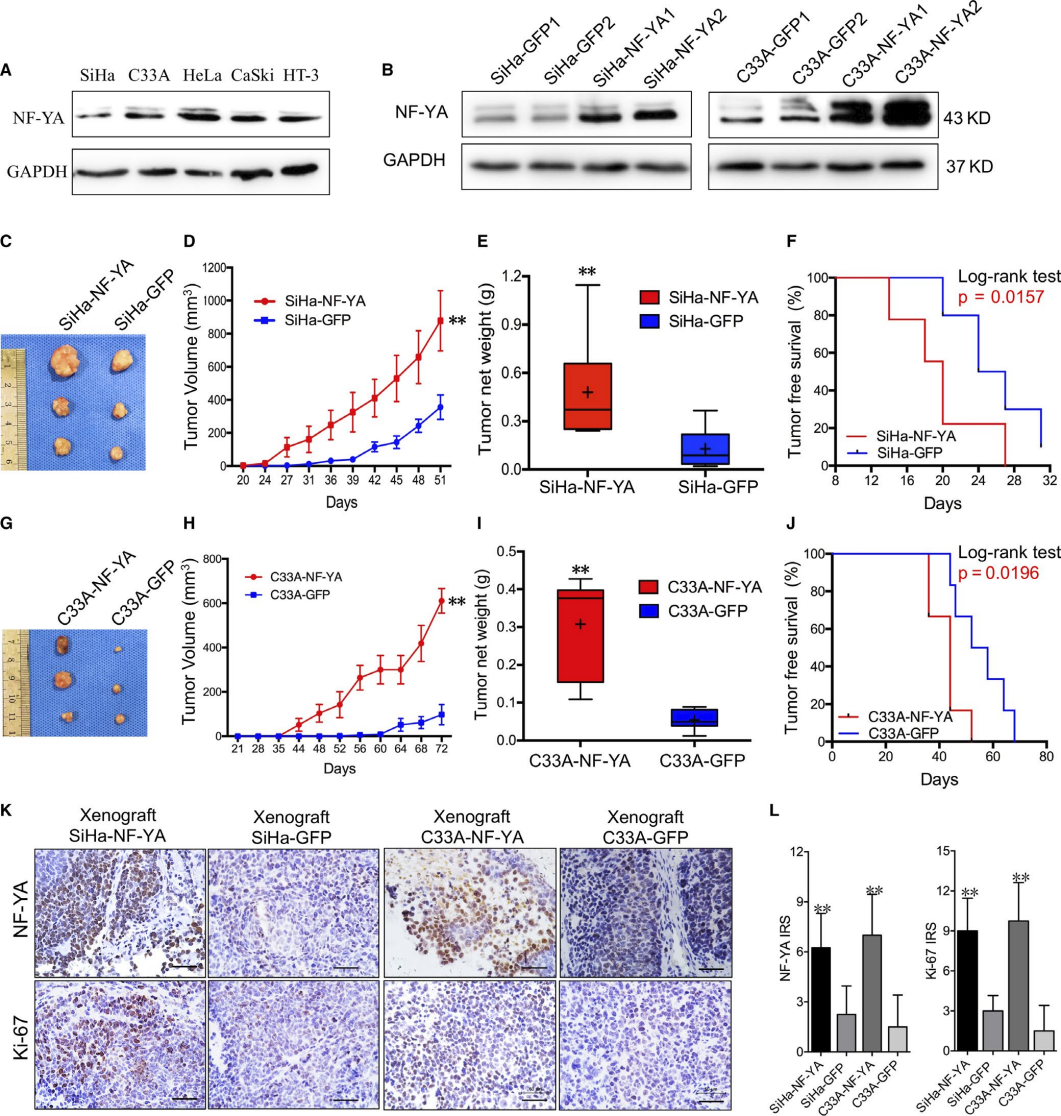
NF‐YA promoted the tumour formation of cervical cancer cells *in vivo*. (A) The expression of NF‐YA protein in cervical cancer cells lines detected by Western blotting. (B) NF‐YA protein was detected in NF‐YA overexpressed SiHa and C33A cells. NF‐YA1 and NF‐YA2 are as for the 2 random sub‐clones of NF‐YA overexpressed cells in SiHa and C33A. GFP1 and GFP2 are also as for the 2 random controls for SiHa and C33A cells, respectively. (C‐F) The tumour growth curve, tumour weight and tumour‐free survival are analysed in SiHa‐NF‐YA and SiHa‐GFP cells with 3 female nude mice for 3 repeats. (G‐J) The tumour growth curve, tumour weight and tumour‐free survival are analysed in C33A‐NF‐YA and C33A‐GFP cells. (K and L) The expressions and IRS values of NF‐YA and Ki‐67 in tumour tissues are shown, scale bar: 50µm. Data were shown as the mean ± SD. The symbols represent the following: ** *P* < 0.01

NF‐YA expression was found in 5 cervical cancer cell lines by Western blotting (Figure 2A). To further investigate the function of NF‐YA in cervical cancer, exogenous expressed NF‐YA cells were obtained in SiHa and C33A cells, which showed the lower level of NF‐YA protein expression (Figure 2B). To determine whether the NF‐YA‐positive cells had a greater capacity to form tumours, a xenotransplantation experiment was performed with 10^4^ NF‐YA‐modified cells (left side) and control cells (right side) subcutaneously inoculated into 4‐ to 6‐week‐old female nude mice (N = 3) for three repeats. The tumour volume, weight and incidence were then monitored every three days. Then, we found that the palpable tumours formed by SiHa‐NF‐YA cells grew more quickly (Figure 2C and D *P* < 0.01) and heavier (Figure 2B, 0.20 ± 0.08g vs. 0.45 ± 0.21g, *P* < 0.05) than that formed by SiHa‐GFP cells. And also, the tumour‐free period exhibited significantly shorter in SiHa‐NF‐YA cells of 12 days as compared to 20 days in SiHa‐GFP cells (*P* < 0.05). The xenotransplantation experiment also performed in C33A‐NF‐YA and C33A‐GFP cells, and similar results of tumorigenic property were found (Figure 2G and J, *P* < 0.05). Furthermore, the expression of cell proliferation marker Ki‐67 was higher in the xenograft tissues formed by NF‐YA‐overexpressed SiHa and C33A cells than that in those formed by control cells, respectively (Figure 2K and L, *P* < 0.05). These results demonstrated that the NF‐YA promotes the tumorigenic property of cervical cancer cells *in vivo*.

### 3.3 NF‐YA‐positive cervical cancer cells shared the higher tumorsphere formation and cell growth *in vitro*

**Figure 3:**
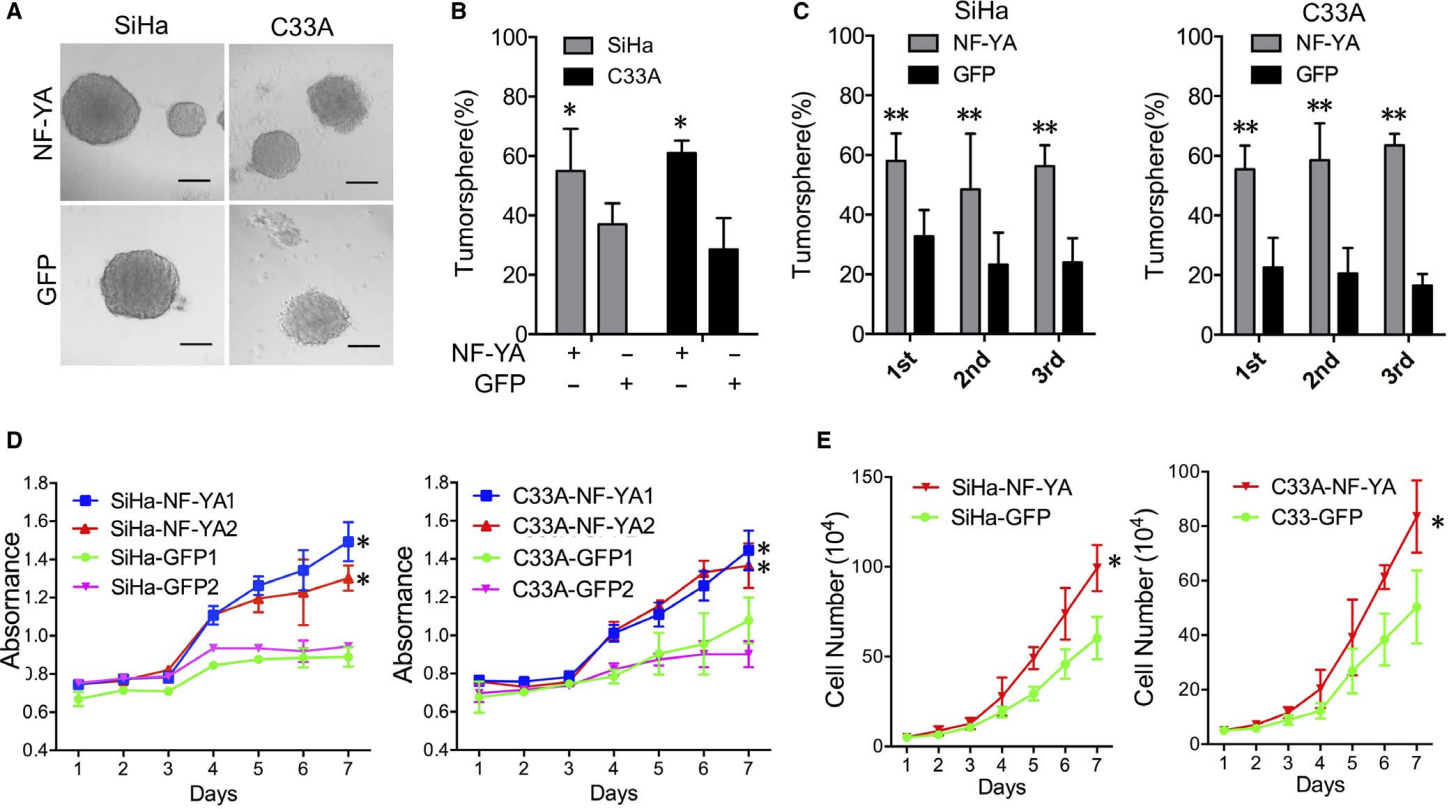
NF‐YA‐positive cervical cancer cells shared the higher tumorsphere formation and cell growth *in vitro*. (A) Representative photographs of tumorspheres and the percentage of tumorspheres formation are shown, scale bar: 50µm. (B and C) The tumorsphere number per 96 cells in serial 3 passages by limiting dilution assay in SiHa‐NF‐YA and C33A‐NF‐YA cells and control cells, respectively. (D and E) Cell viability (MTT) and cell growth assays were determined in SiHa‐NF‐YA and C33A‐NF‐YA cells. Data were shown as the mean ± SD. The symbols represent the following: * *P* < 0.05, ** *P* < 0.01

Previous studies showed that NF‐YA protein was involved in the maintenance of stemness of stem cells by activating multiple other stem cell‐related genes. We focused on the role of NF‐YA in driving cervical CSC characteristics. To assess the ability of self‐renewal, a critical characteristic of CSC *in vitro*, tumorsphere formation ability was valued with cells cultured in the serum‐free medium. As shown in Figure 3A and B, 57.8% and 61.4% of the NF‐YA‐overexpressing SiHa and C33A cells, respectively, generated tumorspheres when plated as a low‐density culture at a density of 100 cells per well in 24‐well plates, while the SiHa‐GFP and C33A‐GFP cells generated much less tumorspheres with the percentage of tumorsphere formation of 38.6% and 24.5%, respectively (*P* < 0.05). Limited dilution method was performed to exclude the effects of cell aggregation occurred in low‐density cultures with the cells cultured at a density of a single cell per well. Upon 3 consecutive passages in culture, the SiHa‐NF‐YA and C33A‐NF‐YA cells generated tumorspheres with a higher efficiency of maintenance than that in GFP cells (Figure 3C, *P* < 0.01), indicating that the overexpression of NF‐YA in cervical cancer cells improved the self‐renewal capacity.

MTT and cell count assays used to value the cell viability and growth showed that NF‐YA overexpressed SiHa and C33A behaved significantly increased cell growth and cell vitality as compared to GFP controls, respectively (Figure 3D and E, *P* < 0.05). To determine whether the cell proliferation was involved in the tumour promotion by NF‐YA, the cell cycle distribution was analysed using FACS. The percentage of SiHa‐NF‐YA cells in S phase increased significantly while a decrease in the percentage of SiHa‐NF‐YA cells in M phase was observed as compared with SiHa‐GFP cells (Figure S1A, *P* < 0.05). A similar effect was observed in C33A‐NF‐YA cells (Figure S1B, *P* < 0.05). Conclusively, NF‐YA promoted the self‐renewal of tumorsphere and cell growth *in vitro*.

### 3.4 NF‐YA‐positive cervical cancer cells shared ability of drug resistance

**Figure 4:**
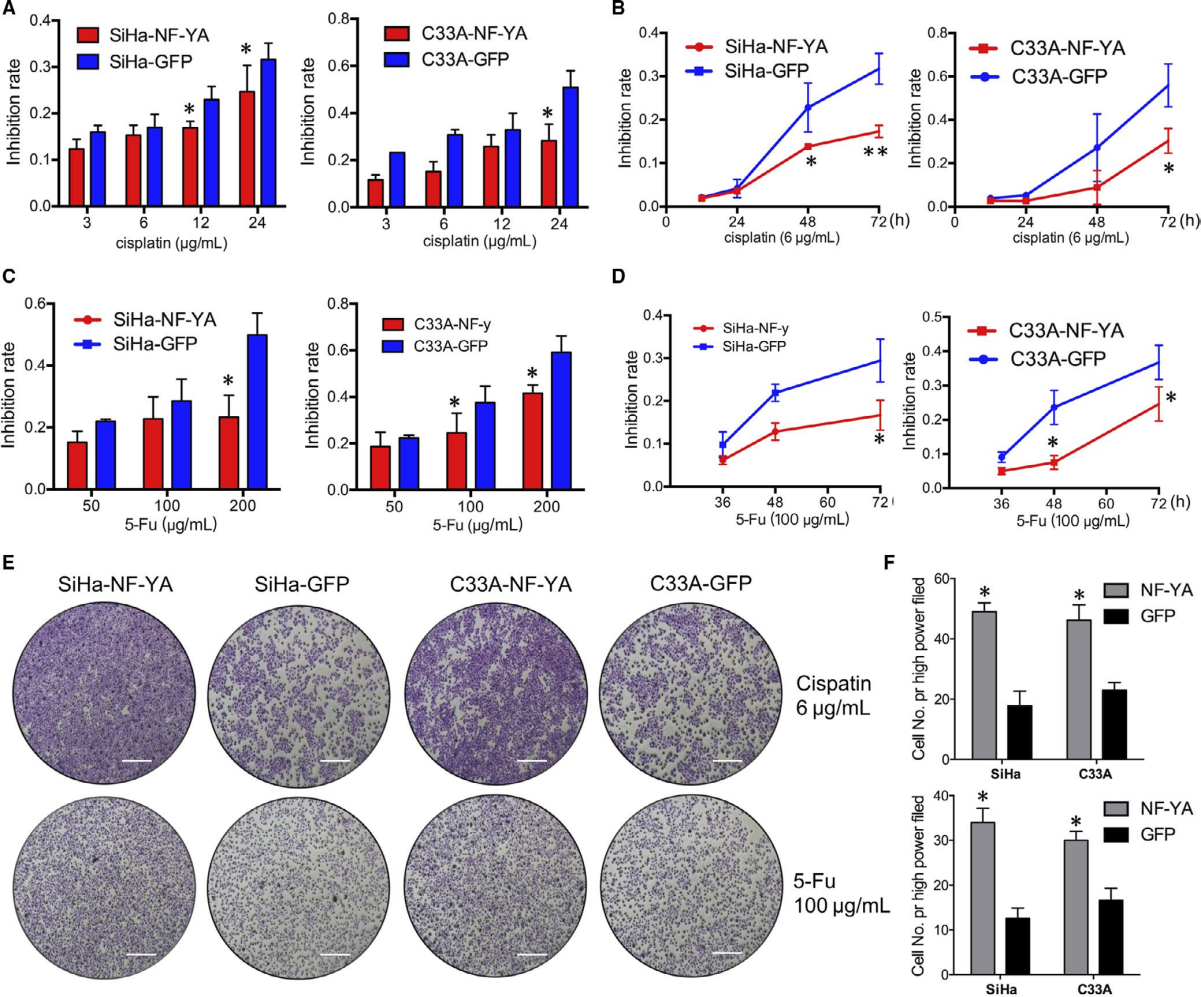
NF‐YA‐positive cells shared the chemical resistance characteristics of CSC. (A and B) Cell viability was measured using an MTT assay after treatment with different concentrations of cisplatin for 24 h and calculated the IC_50_ of different cell groups. (B) Cell viability was measured using an MTT assay after treatment with a constant dose (6 μg/mL) of cisplatin for 1, 3, 5 and 7 days. (C) Cell viability was measured using an MTT assay after treatment with different concentrations of 5‐Fu for 48 h and calculated the IC_50_. (D) Cell viability was measured using an MTT assay after treatment with a constant dose (100 μg/mL) of 5‐Fu for 36, 48, 60 and 72 h. (E and F) Giemsa staining after exposure to constant concentration of 6 μg/mL cisplatin or 100 μg/mL 5‐Fu for 72 h in SiHa‐NF‐YA and C33A‐NF‐YA cells. Data are presented as the mean ± SD of experiments in triplicate and statistically analysed with Student’s *t* test. The symbols represent the following: *P < 0.05, **P < 0.01

The resistance of CSC to current chemotherapeutics is thought to be responsible for cancer recurrence and metastasis. Cells were exposed to the cisplatin, one of the most commonly chemotherapeutic drugs in cervical cancer treatment with different concentrations for 24 hours, and then, the cell viability was detected by MTT assay, and then, the IC_50_ value was calculated. SiHa‐NF‐YA and C33A‐NF‐YA cells showed significantly more resistant to cisplatin with the IC_50_ of 8.40 ± 0.23 μg/mL and 14.03 ± 1.46 μg/mL than the control cells, respectively (Figure 4A, *P* < 0.05). Meanwhile, SiHa‐NF‐YA and C33A‐NF‐YA cells were significantly more resistant to ≥ 48 hours of treatment with cisplatin at a concentration of 6μg/mL compared to control cells (Figure 4B, *P* < 0.05). We also determined that NF‐YA inhibited the sensitivity with the significant decrease of IC_50_ value to another chemotherapeutic drug 5‐FU in both SiHa and C33A cells (Figure 4C and D *P* < 0.05). Additionally, the number of viability cells was counted in 10 random high‐power fields by Giemsa staining after exposure to a constant concentration of 6 μg/mL cisplatin and 100 μg/mL 5‐Fu for 72 hours, suggesting much higher viability both in NF‐YA overexpressed SiHa and C33A cells than that in control cells (Figure 4E and F, *P* < 0.05). All these results above suggested that NF‐YA contributed to the chemoresistance of cervical cancer.

### 3.5 NF‐YA promoted the cell proliferation and tumorigenicity by up‐regulating SOX2 in cervical cancer

**Figure 5:**
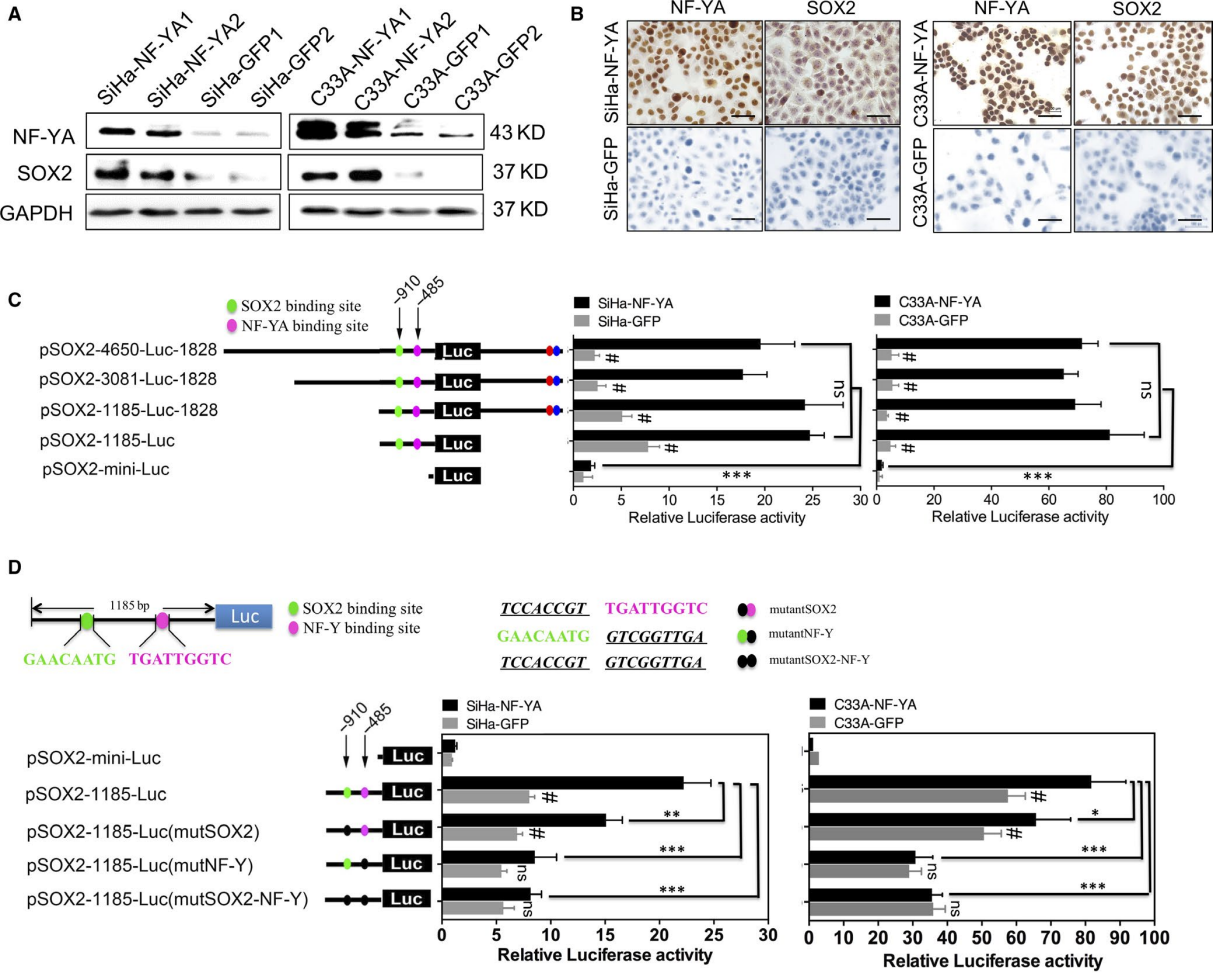
NF‐YA up‐regulated the expression of SOX2 in cervical cancer cells. The expression of SOX2 protein was detected in SiHa‐NF‐YA and C33A‐NF‐YA cells by Western blotting (A) and IHC (B). (C) The SOX2 promoter deleted reporters were constructed, and luciferase activity relative to Renilla activity was detected in SiHa‐NF‐YA, C33A‐NF‐YA cells and controls, respectively. The SOX2 transcriptional activity was expressed relative to pSOX2‐mini‐luc reporter served as the negative control. (D) The diagram of SOX2 promoter deletion containing the possible cis‐acting elements and the NF‐YA and SOX2 binding sites. The luciferase activity of the SOX2 promoter mutations (green dot: SOX2 binding site; pink dot: NF‐Y binding site; black dot: mutant of SOX2 or NF‐Y binding site) was detected in NF‐YA‐overexpressing SiHa and C33A cells. Data are presented as the mean ± SD of experiments in triplicate and statistically analysed with Student’s *t* test. The symbol *, *P* < 0.05, ** and ***, *P* < 0.01, while ns indicates no statistical difference

All these results above suggested that NF‐YA could maintain the characteristic of cervical cancer CSC by increased the properties of tumorigenicity, cell growth, self‐renewal and chemoresistance. In our previous study, SOX2 protein has been identified as a marker of cervical CSC. Here, we found that NF‐YA up‐regulated the expression of SOX2 protein detected by Western blotting and IHC (Figure 5A and B). Combining with the trans‐activation function of NF‐YA to the SOX factors, we analysed the SOX2 promoter, containing 4650bp and 1828bp through two sides of CDS region, and constructed several deletions fused to pGL3 basic luciferase reporter plasmid (Figure 5C). The luciferase activity detected by dual‐luciferase reporter assay in NF‐YA overexpressed SiHa and C33A cells was significantly higher than that in control cells when containing −1185bp region regardless of whether there was −1828 downstream or not (Figure 5C, *P* < 0.05).

Additionally, two binding sites (SOX2 and NF‐Y) were found in the candidate region of SOX2 promoter based on the Web Promoter Scan Service and TF SEARCH online software. Then, 2 mutations in SOX2 binding site (GAACAATG to TCCACCGT, pink site) and in NF‐Y binding site (TGATTGGTC to GTCGGTTGA, green site) of plasmid pSOX2‐1185‐luc were constructed (Figure 5D). The luciferase activity of both the 2 mutations was significantly inhibited compared with pSOX2‐1185‐Luc reporter in NF‐YA overexpressed cells. Of note the mutation of NF‐Y binding site resulted in a significant decrease in transcriptional activity in NF‐YA overexpressed cells to the level of that in control cells (Figure 5D, *P* < 0.05). These results suggested that CCAAT/ATTGG box upstream of SOX2 promoter was indispensable for the transcription of SOX2 by NF‐YA.

### 3.6 NF‐YA up‐regulated the expression of SOX2 through directly binding to the CCAAT/ATTGG box of SOX2 promoter in cervical cancer cells

**Figure 6:**
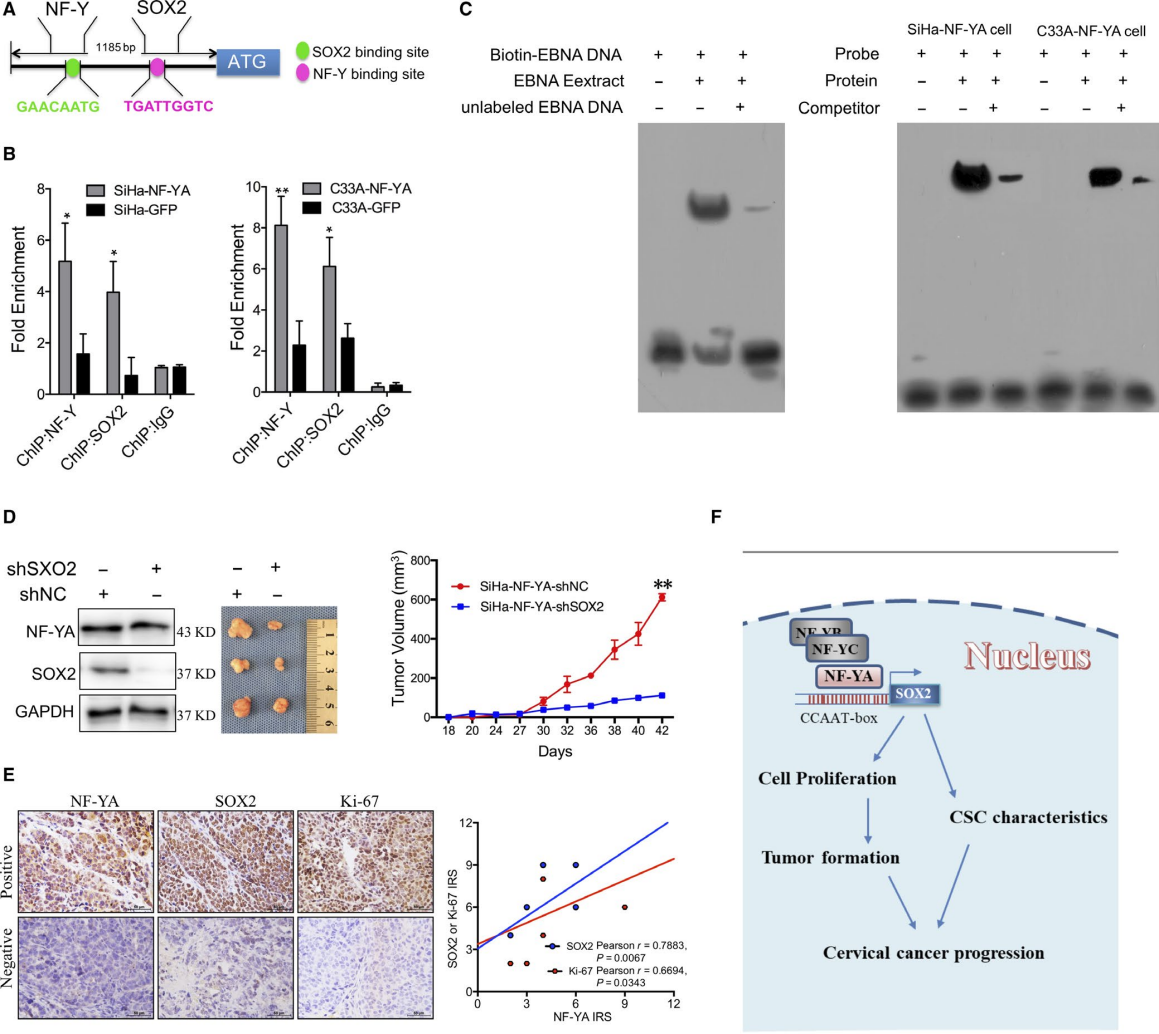
NF‐YA specifically bound to CCAAT/ATTGG box upstream of the SOX2 promoter in cervical cancer cells. (A) The diagram of SOX2 promoter upstream with the binding sites of NF‐YA factor. (B) The qChIP assay is shown in NF‐YA‐overexpressing SiHa and C33A cells. The cell lysate was immunoprecipitated by NF‐YA or SOX2 antibodies and IgG antibody (as the negative control). (C) EMSA was performed by incubating nuclear lysate from NF‐YA‐overexpressing SiHa and C33A cells with biotin‐labelled DNA fragment containing an NF‐YA binding sequence and the EBNA extract served as the positive control. (D) SOX2 was silenced by specific shRNA targeting the SOX2 CDS region in NF‐YA‐overexpressing SiHa cells, and the tumour growth curve was shown. (E) The expression of NF‐YA, SOX2 and Ki‐67 was detected by IHC in 10 SCC samples, and the relationship was determined using the Pearson correlation. (F) Proposed model of the NF‐YA‐mediated up‐regulation of SOX2 in cervical progression. Data are presented as the mean ± SD of experiments in triplicate. The symbols represent the following: *, *P* < 0.05; **, *P* < 0.01

Next, we needed to determine whether the expression of SOX2 was mediated by NF‐YA directly binding to the SOX2 promoter region of CCAAT/ATTGG box. The schematic map of the SOX2 promoter upstream of the SOX2 and NF‐Y binding site is shown in Figure 6A. ChIP assay demonstrated that SOX2 and NF‐YA protein physiologically binds directly to the cis‐element in SiHa‐NF‐YA and C33A‐NF‐YA cells, respectively (Figure 6B, *P* < 0.05). Additionally, EMSA was then employed to assess whether the nuclear protein lysate from the SiHa‐NF‐YA and C33A‐NF‐YA cells binds to the probe sequence containing the CCAAT/ATTGG box. As shown in Figure 6C, we detected a strong band in the group of probe and protein in SiHa‐NF‐YA and C33A‐NF‐YA cells. Additionally, biotin‐EBNA DNA and EBNA extract supplied by the reagent kit were carried out as the positive control. In order to further confirm SOX2 was up‐regulated by NF‐YA, the SOX2 protein in SiHa cells overexpressed NF‐YA was silenced, and xenograft assay showed that the tumour formation ability was inhibited. This suggests that silenced SOX2 could reverse the tumour growth promoted by NF‐YA (Figure 6D, *P < 0.05*). At last, the IHC assay of cervical cancer tissues from patients (N = 10) determined the positive correlation between NF‐YA and Ki‐67/SOX2 (Figure 6E, *P* < 0.05). Also, the TCGA database showed that NF‐YA mRNA was positive related both to the cell proliferation marker Ki‐67 and pluripotency marker SOX2 (Figure S2, *P* < 0.05). These findings suggested that NF‐YA up‐regulated the expression of SOX2 in cervical cancer cells by directly binding to the NF‐Y binding site (CCAAT/ATTGG box) upstream of the SOX2 promoter (Figure 6F).

## 4 DISCUSSION

NF‐Y, also known as the CCAAT‐binding factor, is an evolutionarily conserved transcription factor composed of three subunits: NF‐YA, NF‐YB and NF‐YC. It binds to CCAAT motif in the proximal promoter region to induce gene expression.^29^ NF‐YA which is considered as the limited regulatory subunit is required for the complex assembly and DNA binding.^30^

NF‐Y has been reported to be involved in the embryo‐genesis and tumour progression.^31^ Recent observations highlight that NF‐Y involved in the maintenance of CSC characteristics of several type of cancers such as oral cancer, hematopoietic stem cell and embryonic carcinoma.^20^, ^25^, ^32^ NF‐YA also involved in the cell proliferation, metastasis and other malignant biological in different types of carcinomas. In breast cancer, NF‐YA is associated with a proliferative signature, signal loss of epithelial features, acquisition of EMT and more aggressive behaviour and also has worst clinical outcomes.^33^ Also, NF‐YA contributes to tumour invasion and angiogenesis through EZH2‐STAT3 signalling in human melanoma cells.^34^ In hepatocellular carcinoma, NF‐YA repressed by ZHX2 inhibited the activation of MDR1 transcription and, in doing so, enhances the effects of chemotherapeutics.^35^ However, in human embryonal carcinoma cells, NF‐YA significantly reduced the cell growth and cell pluripotency by the decrease in the level of the pluripotency marker SOX2.^24^ Whether and how it participates in the process of cervical carcinogenesis remains obscure. Here, IHC analysis has revealed the gradually high expression of NF‐YA from NC to HISL and then to SCC, indicating that NF‐YA might promote the progression of cervical cancer. Additionally, NF‐YA promoted the cell proliferation *in vivo* and *in vitro* by tumour xenograft, cell growth and cell viability assays. The CSC characteristics of self‐renewal and chemoresistance abilities were also increased. Here, we firstly identified that NF‐YA maintains the stemness and tumorigenic properties of cervical cancer cells.

NF‐Y has been reported to transcriptional activate the expression of SOX genes, including SOX3, SOX9 and SOX18 mediated, at least in part, by direct binding to CCAAT boxes.^26^, ^27^, ^28^ As the target gene, SOX2 has been confirmed being repressed by NF‐YA to decrease the proliferation and pluripotency in embryonic carcinoma.^24^ Here, we found a new binding site for NF‐YA in the SOX2 promoter, resulting to the transcription in cervical cancer cells. Meanwhile, SOX2 has been previously identified as the key factor in several types of CSC,^36^, ^37^ including cervical cancer.^16^ We previously have isolated the SOX2‐positive cervical CSC from SiHa and C33A, which exhibited the major characteristics of CSC, including self‐renewal, differentiation and tumour progression properties.^17^

To further define the cis‐regulatory elements of SOX2 promoter bound by NF‐YA, the TFSEARCH database was used to identify a putative CCAAT motif, which was the binding site of NF‐YA. Mutagenesis of this motif demonstrated that CCAAT box plays a crucial functional role in the regulation of the SOX2 promoter. Gel mobility shift analysis and ChIP assay demonstrated that the CCAAT box motif could bind the transcription factor NF‐Y *in vitro and in vivo*. As the activator of SOX2, high expression of NF‐YA in cervical cancer was positive related to it and promoted the stemness of cervical cancer cells, suggesting that NF‐YA might be another biomarker or regulator of cervical CSC. However, whether there are other target genes of pathway regulated by NF‐YA to promote the cell proliferation and tumorigenicity should to be further explored.

In conclusion, NF‐YA highly expressed in cervical cancer, promoted the cell growth *in vitro* and *in vivo* and maintained the cervical CSC characteristics by driving SOX2 expression. This study might provide an important insight into the biology of CSC and identified a potential target for intervention of cervical cancer.

## Conflict of Interest

The authors declare that they have no conflict of interest.

## Supporting information

Fig S1

Fig S2

Table S1

## Data Availability

The data used to support the findings of this study are available from the corresponding author upon request.
