# Supplementary figures and images for: NF‐YA promotes the cell proliferation and tumorigenic properties by transcriptional activation of SOX2 in cervical cancer
Source: J Cell Mol Med. 2020 Sep 20;24(21):12464–75. doi: 10.1111/jcmm.15777 (PMC7686972; doi:10.1111/jcmm.15777)

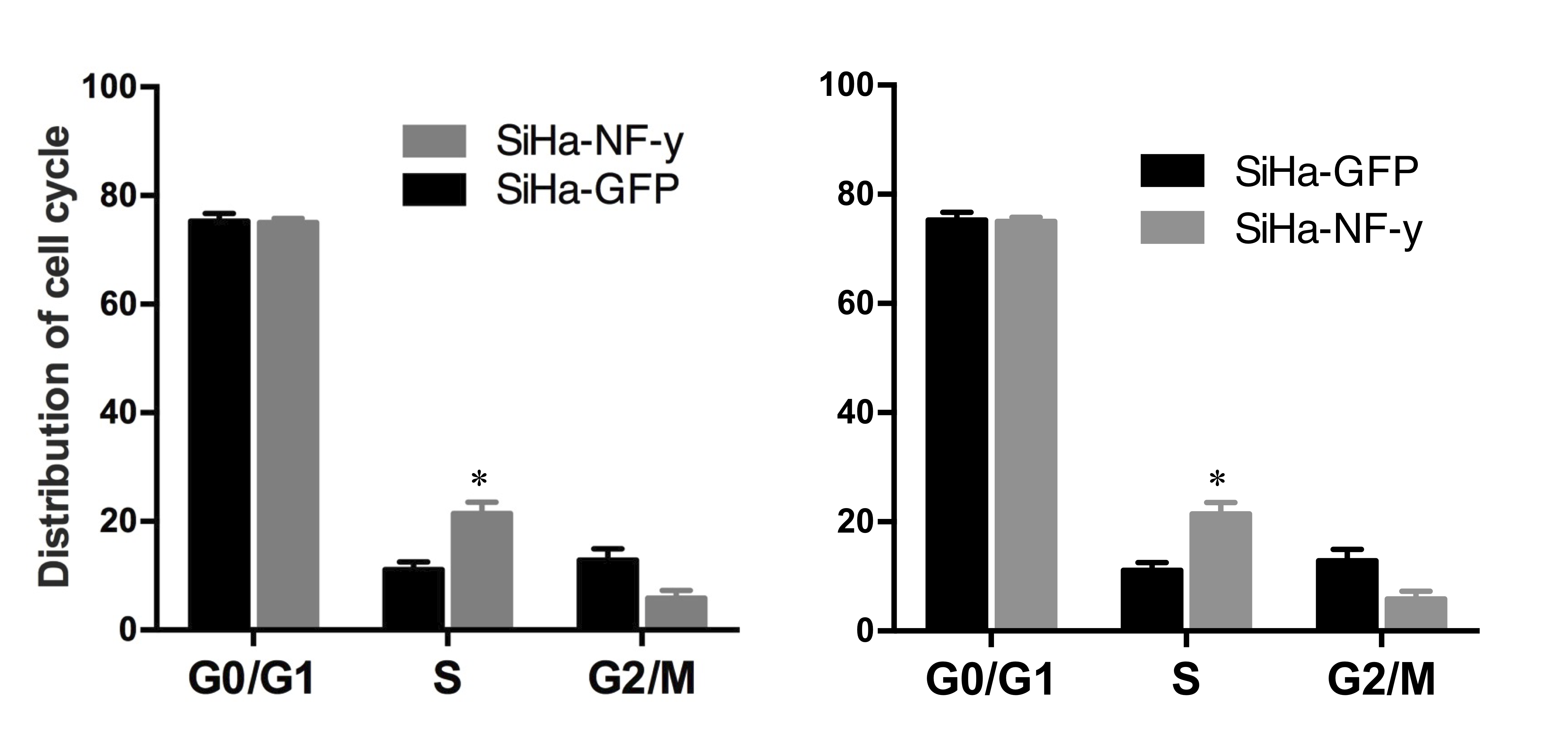

Supplement: Supplementary file 1 — Fig S1 [file JCMM-24-12464-s001.tiff]

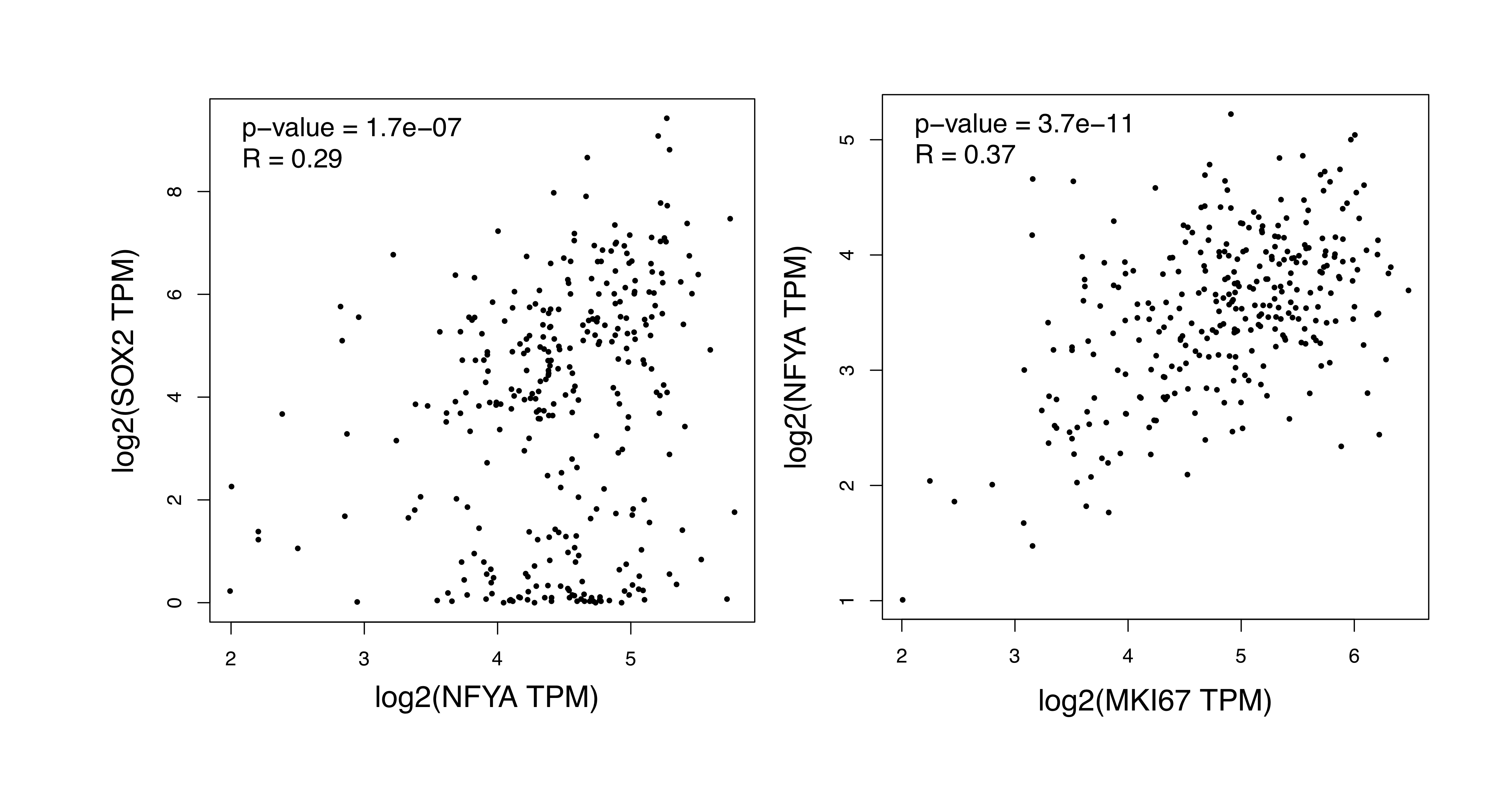

Supplement: Supplementary file 2 — Fig S2 [file JCMM-24-12464-s002.tiff]
